# 65993 Peptide Conjugated Hollow, Degradable Nanoparticles Bind to Exposed Hyaluronic Acid for the Prevention and Treatment of Osteoarthritis

**DOI:** 10.1017/cts.2021.762

**Published:** 2021-03-30

**Authors:** Marcus Deloney, Parssa Garoosi, Blaine Christiansen, Alyssa Panitch

**Affiliations:** University of California Davis

## Abstract

ABSTRACT IMPACT: Our research would be the first therapeutic to both prevent and treat osteoarthritis - helping 27 millions U.S. citizens alone immediately. OBJECTIVES/GOALS: Our objective is to conjugate hyaluronic acid binding peptides (HABP) to anionic hollow nanoparticle (hNP), and allowing the HABP-hNP complex to penetrate into osteoarthritic cartilage, bind to exposed HA, prevent further degradation, and restore the compressive strength of articular cartilage. METHODS/STUDY POPULATION: N-isopropyl acrylamide, 2-acrylamindo-2-methyl-1-propanesulfonic acid, N,N’-bis(acryoyl)cystamine, and Acrylic Acid, in fluorescent batches rhodamine b isothiocyanate (RBITC), were polymerized via precipitation reaction. HA binding peptide, GAHWQFNALTVRGSG-Hydrazide (GAH-Hyd), was covalently bonded to the hNP using DMTMM chemistry. The reaction was halted by diluting the solution 10:1 with milliQ water and purified using tangential flow filtration. The dynamic viscosity of the six treatments were analyzed in a 70 kDa HA. Using a rheometer (Discovery HR-3) with a 20 mm parallel plate geometry, TA Instruments, New Castle, DE), a frequency sweep (0.01 -1000 Hz, 2.512 Pa) was conducted to measure the storage modulus of each solution. RESULTS/ANTICIPATED RESULTS: GAH-Hyd was successfully conjugated to the surface of the hNP and zeta-potential shows a significant increase in surface charge from -21.41 mV for unconjugated hNP to -8.94 mV for 65 GAH conjugated hNP, confirming conjugation. The hNPs need 65 ±10 GAH per nanoparticle to significantly bind to HA, shown by increasing the dynamic viscosity of the solution. The minimum concentration of 65 GAH-hNP required to significantly bind to HA is 313 µM. These data from our study display the ability to functionalized the surface of polymeric hNPs with site specific peptides and their ability to bind to diseased tissue. We expect the GAH-hNP system will restore the compressive strength of OA cartilage and prevent further HA degradation in ex vivo aggrecan depleted cartilage plugs. DISCUSSION/SIGNIFICANCE OF FINDINGS: Binding to exposed HA within the ECM of cartilage protects the HA from further degradation, halting the progression of OA. 65 GAH-hNP binds to HA at a 313 µM. Our system can be translated and used to treat a multitude of conditions by conjugating tissue specific peptides to the surface of our hNPs and delivery site specific therapeutics to diseases tissue.

